# Odontogenic tumors: A collaborative study of 218 cases diagnosed over 12 years and comprehensive review of the literature

**DOI:** 10.4317/medoral.19157

**Published:** 2014-09-30

**Authors:** Ahmet-Ercan Sekerci, Sinan Nazlım, Meryem Etoz, Kemal Denız, Yasin Yasa

**Affiliations:** 1 Assistant Professor, Department of Oral and Maxillofacial Radiology, Faculty of Dentistry, Erciyes University, Kayseri, Turkey; 2 Department of Pathology, School of Medicine, Erciyes University, Kayseri, Turkey; 3 Research Assistant, Department of Maxillofacial Radiology, Faculty of Dentistry, Erciyes University, Kayseri, Turkey; 4 Research Assistant, Department of Maxillofacial Radiology, Faculty of Dentistry, Ataturk University, Erzurum, Turkey

## Abstract

Objectives: The objective of this study was to analyze the frequency and distribution of odontogenic tumors (OTs) in the Cappadocia region of Turkey, and to compare the findings with those reported in the literature. Study Design: The records of the Oral and Maxillofacial Surgery and Pathology Departments at Erciyes University, with histologic diagnosis of odontogenic tumors (based on the World Health Organization classification, 2005), over a 12-year period, were analyzed. The relative frequency of different types of tumors was also analyzed and compared with the literature. Results: OTs in the present study constituted 2.74% of all the 7,942 registered biopsies. A total of 218 cases of OTs were collected and reviewed. Of these, (94.04%) were benign and (5.96%) were malignant. The mandible was the most commonly affected anatomic location, with 170 cases (77.9%). Ameloblastoma with a predilection for the posterior mandible was the most frequent odontogenic tumor (30.28%), followed by keratocystic odontogenic tumor (19.5%), odontoma (13.4%), and odontogenic myxoma (8.5%). Conclusions: OTs are rare neoplasms and appear to show geographic variations in the world. In Cappadocia, Turkey, they are more common in the mandible, with ameloblastoma followed by keratocystic odontogenic tumors with the incidences observed in the present study being similar to those of previous studies from Asia and Africa, and in contrast to those reported from American countries.

** Key words:**Odontogenic tumors, WHO classification, prevalence, jaws.

## Introduction

Odontogenic tumors (OTs) constitute a heterogeneous group of lesions, arising from the tooth-producing tissues or its remnants (1). From a biological point of view, some of these lesions represent hamartomas with varying degrees of differentiation, while the rest are benign or malignant neoplasms with variable aggressiveness and potential to develop metastasis (2). OTs are rare lesions of the mandible and maxilla that must be considered as a part of the differential diagnosis of lesions that occur in the jaws (3). In humans, tumors of the odontogenic tissues are comparatively rare, comprising about 1% of all jaw tumors (4).

The first internationally accepted classification system for OTs was published in 1971 by the World Health Organization (WHO), which was reviewed and updated in 1992 and in 2005 (2).

Knowledge of their epidemiology and clinical presentation is essential, and retrospective studies have been carried out in Asia (5-16), Africa (17-30), Europe (31-33), North America (3,34-40), and South America (41-49) to describe these lesions. The geographic distribution of these lesions is variable, mainly because of high genetic and cultural diversity (41). Their etiology is unknown and the majority develops without an apparent cause (30).

It is very important to form a set of criteria such as sex, age, and location of lesion, in the management of OTs. Epidemiological studies are crucial because they allow us to establish more precisely the occurrence of OTs in different populations, which in turn helps in the making of a provisional diagnosis and further planning of the biopsy based on the clinical and radiographic features. It also aids in patient counseling and scheduling of treatment (14).

Furthermore, there is no information available in the English-language literature on the relative frequency of OTs in Turkey or, particularly, in the Cappadocia region, according to the 2005 WHO classification. The purpose of the present study was to determine the relative prevalence of different types of OTs and to determine the relative incidence of different OTs in the world population through analysis of published studies and statistics, and by comparing these data with each other and with those already reported in the literature.

## Material and Methods

In the present study, the surgical histopathology records of the Departments of Oral Pathology, Faculty of Medicine and Oral and Maxillofacial Surgery, Faculty of Dentistry, Erciyes University were reviewed retrospectively from August 2001 to January 2013. They were tabulated and systematically analyzed to assess the frequency of occurrence based on age, sex, anatomical site and type.

Hematoxylin and eosin stained sections were reviewed to confirm or to correct a previous histological diagnosis according to the criteria suggested for the 2005 WHO classification. The independent opinions of two examiners were compared to reach the final diagnosis and, in cases of doubt, we consulted another expert oral pathologist to obtain a diagnosis by consensus.

A total of 218 cases of OTs were collected and reviewed. The literature was retrieved using Pubmed in English only. Recurrent tumors were considered as a single case. With regard to site distribution, the maxilla was divided into three anatomic regions: anterior, premolar and molar; and the mandible was divided into three anatomic regions: anterior, premolar, molar/ ramus. Data were analyzed using SPSS software (version 11.5; SPSS, Inc, Chicago, IL).Tests were considered statistically significant when the *p*-value was <0.05.

## Results

**Figure 1:**
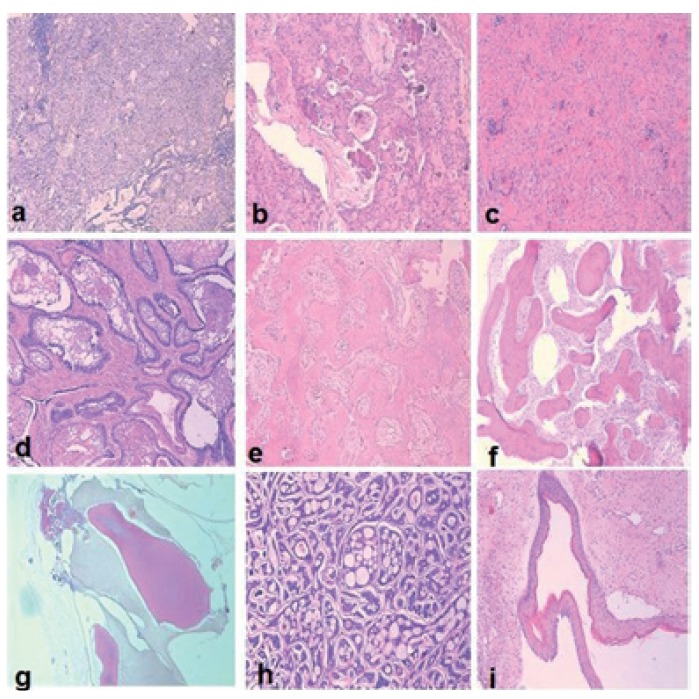
Examples of odontogenic tumors diagnosed with histopathological examination. a) Adenomatoid odontogenic tumor, H&Ex100; b) Calcifying epithelial odontogenic tumor, H&Ex100; c) Odontogenic fibroma, H&Ex100; d) Ameloblastoma, H&Ex100; e) Cementoblastoma, H&Ex100; f) Komplex odontoma, H&Ex100; g) Kompound odontoma, H&Ex40; h) Ameloblastic carcinoma, H&Ex100; i) Keratocystic odontogenic tumor, H&Ex100.

**Figure 2:**
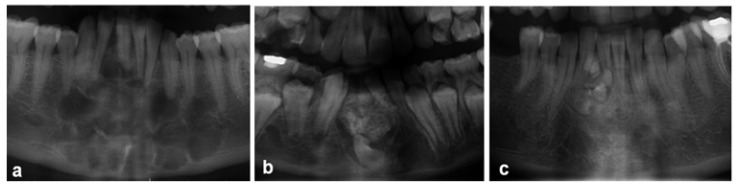
Examples of cropped panoramic radiographs of patients with odontogenic tumors: a) Odontogenic myxoma, b) Ameloblastik fibro-odontom; c) Odontoma.

From total of 7,942 oral and maxillofacial biopsies registered during the 15-year period from 1998 to 2013, 218 cases of odontogenic tumors were found. The most frequent lesion was ameloblastoma (AME) (30.28 %), followed by keratocystic odontogenic tumor (KCOT) (26.15 %) ( Table 1). The proportion of benign to malignant lesions was 15.8:1. Taken together, AME, KCOT, odontoma (OD), and calcifying epithelial odontogenic tumour (CEOT) corresponded to nearly 78% of the cases. There were no statistically significant differences between the ameloblastoma and keratocystic odontogenic tumor groups. In male patients, KCOT followed by AME and calcifying epithelial odontogenic tumor (CEOT) were the most common lesions; in female patients, AME, followed by KCOT and OD, were the most frequent OTs (data shown in  Table 1). (Examples of odontogenic tumors diagnosed with histopathological examination were shown in figure 1. Examples of cropped panoramic radiographs of patients with odontogenic tumors were shown in figure 2).

**Table 1:**
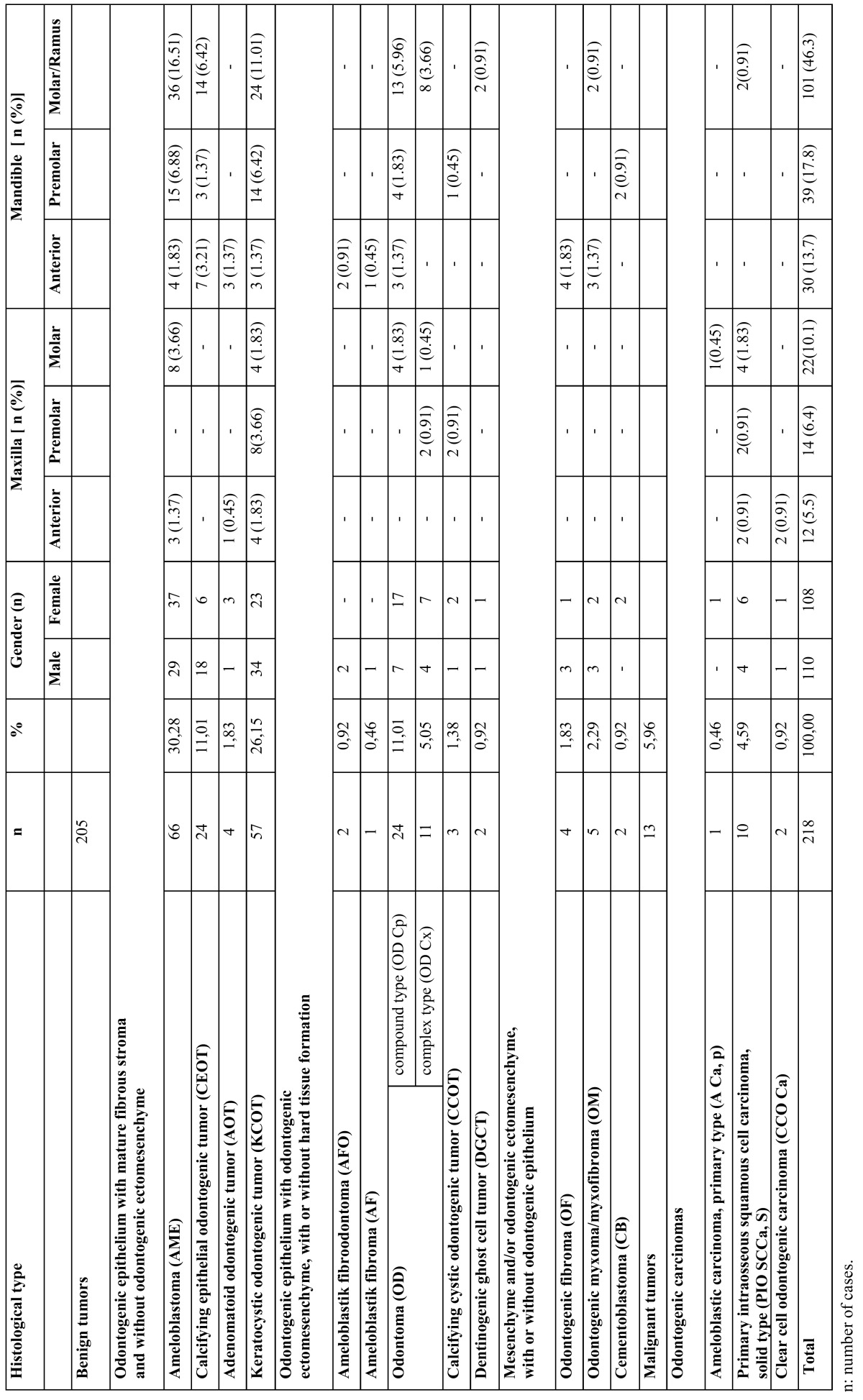
Frequency, gender, and site distribution of 218 odontogenic tumors of patients at two centers.

The other tumors comprised less than 6% of the series. An almost equal gender distribution was observed; with a slight predominance of males (the sample comprised 110 (50.5%) males and 108 (49.5%) females). Statistical analysis revealed no significant difference in the distribution of OT in relation to gender. AME was the only benign tumor found in patients over than 80 years of age. The age of the patients ranged from 10 years to 84 years, with a mean age of 34.52 years. The majority of cases were distributed between the age of 20 and 49 years with a peak incidence in the fourth decade of life ( Table 2).

**Table 2:**
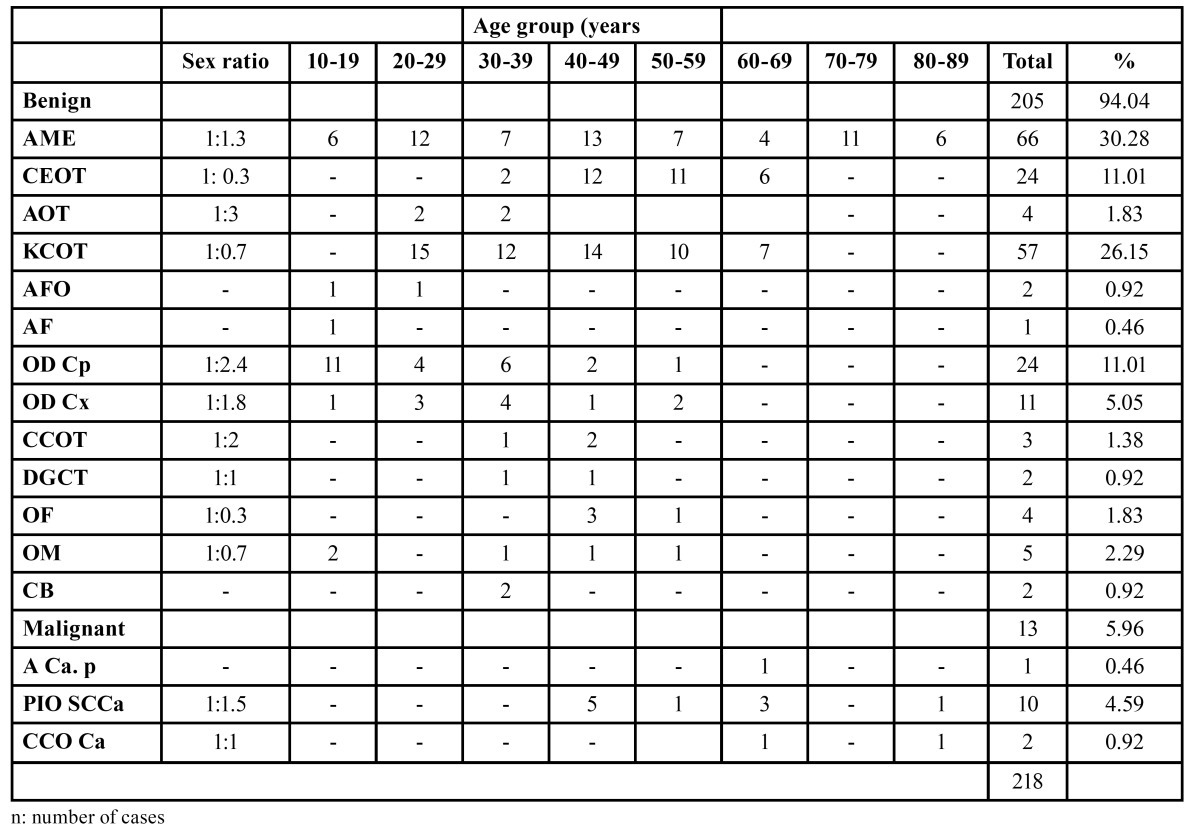
Distribution of odontogenic tumors in different age groups.

The anatomical sites of all cases are also presented in  Table 1. In general, the mandible was the most frequently affected site, corresponding to 77.9 % of the cases, while the maxilla was affected in 22.1% of the cases. The most frequently affected area was the mandibular molar/ramus segment, mainly by AME. The youngest patient who presented with the lesion was 10 years old and the oldest was 84 years old. The single case of ameloblastic fibroma affected a 14-year-old male patient. The lesion was located in the anterior mandible. There were 2 cases of dentinogenic ghost cell tumor (DGCT), which is a new entity according to the 2005 WHO classification.

Malignant OT, ameloblastic carcinoma primary type (A Ca, p), primary intraosseous squamous cell carcinoma (PIO SCCa, S), and clear cell odontogenic carcinoma (CCO Ca) were found more frequently in the maxilla. In the upper jaw, PIO SCCa, S was the most common lesion, mainly observed in the molar region, followed by CCO Ca, mainly in the anterior region. Most malignant OTs also predominantly occurred in patients older than 40 years ( Table 2).

## Discussion

The fact that most OTs remain painless throughout the course of the disease is the main reason that patients do not present until the tumors have reached enormous sizes (17). Knowing the frequency and basic clinical features of OTs is important because this allow us to establish more precisely the expression of these lesions in diverse populations, which in turn helps to identify the groups at risk and possible factors associated with their development, as well as to develop more precise differential diagnoses (2).

In the present study, the relative frequency of odontogenic tumors was 2.74 % of the total biopsied specimens recorded between August 1998 and January 2013. This incidence is similar to what has been reported in other studies, as they represent less than 3% of oral and maxillofacial specimens studied in North American (35,39) (1.55%), South American (41,42,44,45) (1.82%), and European series (32,33) (0.74%). On the other hand, in Asia and Africa OT comprise from 3.9% to 9.6% of all oral lesions (8,9,20), although an Iranian series (7) had a frequency of 1.9%.

This study confirms that benign tumors (94.4%) are the most frequently seen OT; however, malignant OT represented 5.6% in the present series. This frequency of malignant tumors is only similar to those reported in China (8,12), but it is higher than that those published in most other series (23,39,40,42,44-48).

In studies using the new 2005 WHO classification, the most frequent OTs follow the sequence: ameloblastomas (30.28%), KCOT (26.15%), and odontomas (16.06%) ( Table 3). Studies that employed the 1992 classification usually reported ameloblastomas as the prevalent OT, followed by odontomas and odontogenic myxomas ( Table 4). This regional difference has been attributed to the asymptomatic nature of many odontomas and consequent lack of professional management rather than genetic or environmental differences among these populations (19,20,35,44). The present study found ameloblastoma to be the most frequent odontogenic tumor, accounting for 30.28%, followed by KCOT (26.15%), odontoma (16.06%), and CEOT (11.01%). There were no statistically significant differences among the ameloblastoma and KCOT and odontoma groups. These results are comparable with the corresponding data reported by Jing *et al*. (10), Tawfik *et al*. (17) and Osterne *et al*. (46). Ameloblastoma is reported to be the most frequent lesion in Chinese, Egyptian and Brazilian populations, followed by KCOT and odontoma.

**Table 3:**
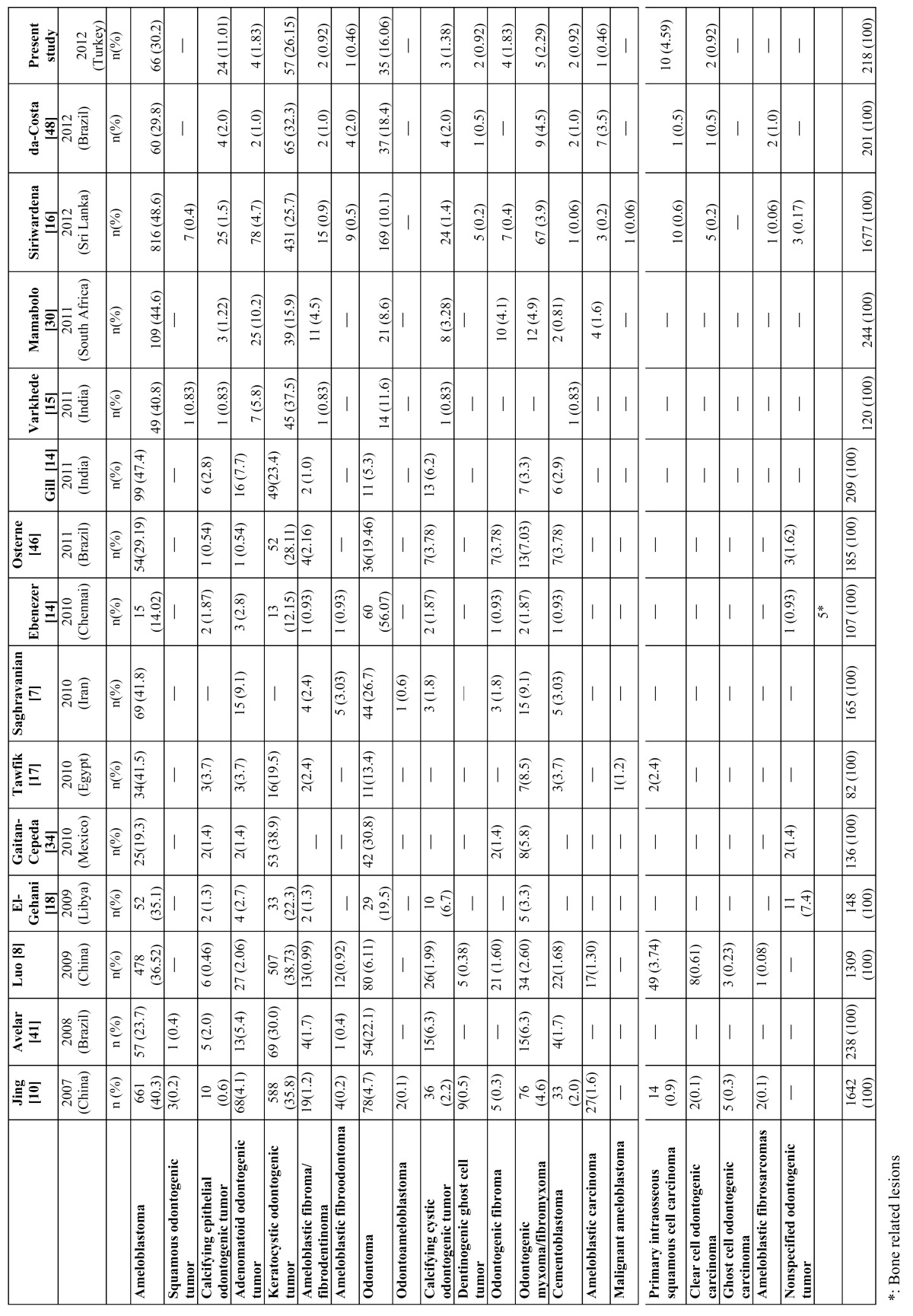
Distribution of odontogenic tumors by diagnosis (2005 WHO classification).

**Table 4:**
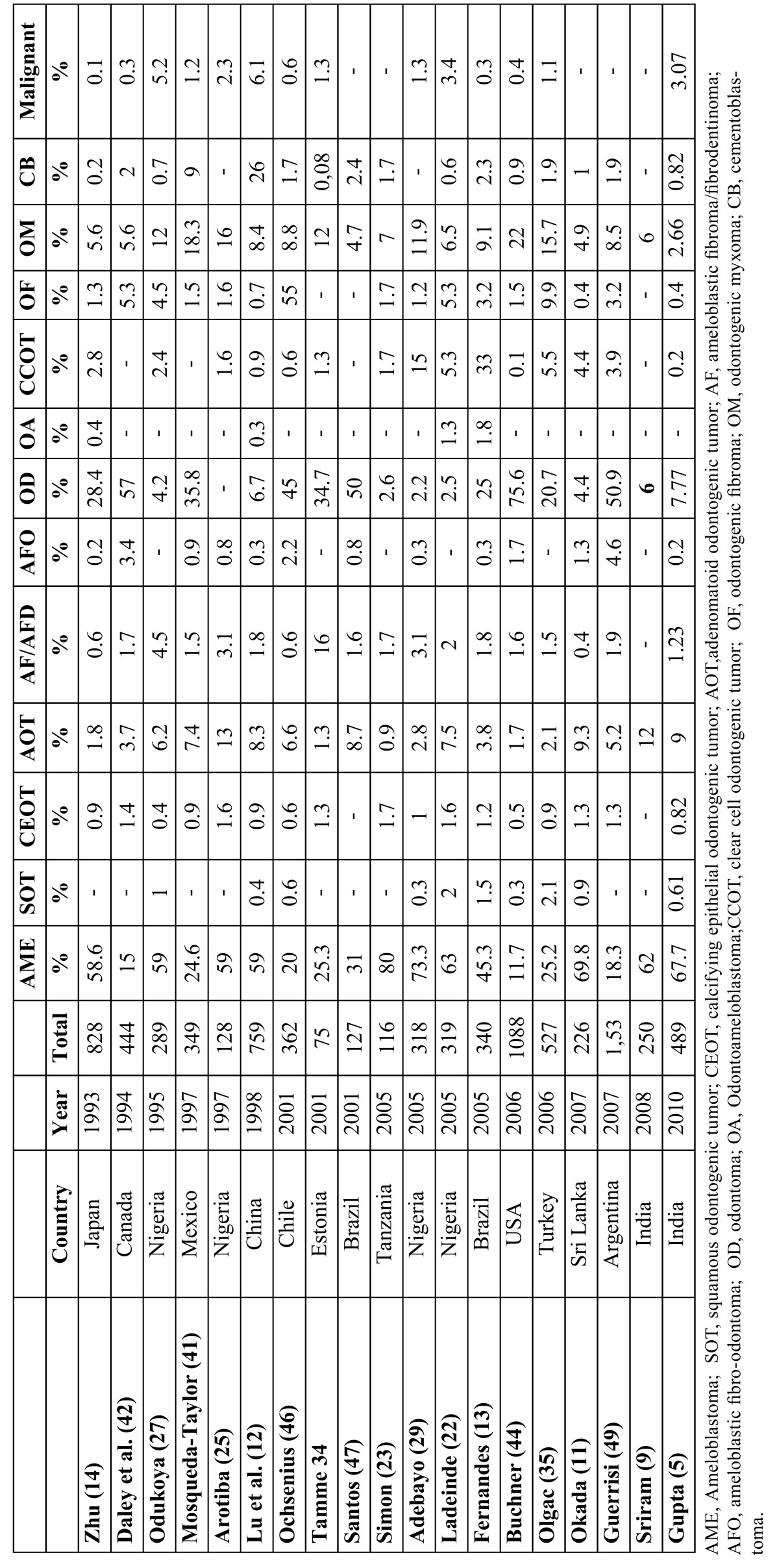
Comparison of prevalence studies around the world (1992 WHO classification).

The high frequency of AME and low frequency of odontoma are consistent with data from Tanzania (21), Nigeria (20,25,44), and Sri Lanka (11) whereas studies from the USA (35), Canada (22), Chennai-India (14) and Estonia (9), stated that odontoma occurs more frequently. These discrepancies in the number of odontomas being less in their populations in comparison with others are probably the result of geographic variation, but it should be mentioned that the incidence of odontoma in some countries was probably underestimated due to the unique clinical features of this tumor and insufficient hospital management (32). Most of these tumors exhibit self-limited growth and do not cause clinical symptoms. Many patients do not think it is necessary to consult a general dentist or even an oral and maxillofacial surgeon. Treatment in many cases was performed in the office and the cases were not recorded or sent for histopathological confirmation (9,17).

The present study showed that AME was the most frequent OT, occurring mainly in the posterior region of the mandible. This is similar to other studies reported from Japan (14), Iran (7), India (14,15), Srilanka (16), Africa (17,18,20,23,25,30), Turkey (33), Hong Kong (13), and China (10,12), but in contrast to those reported from Canada (40), Chile (44), USA (35), Chennai (14) and Mexico (34,39), where odontoma is reported as the most common odontogenic tumor. This also strengthens the belief that ameloblastomas are more common in Asians and Africans compared with Caucasians. A study form Brazil reported that ameloblastoma diagnosis exhibits no gender predilection (43). Reichart *et al*. (49) in an extensive review of all of the cases reported in the literature, reported the average age of initial diagnosis in industrialized countries to be 39.1 years compared with 27.7 years from developing countries. Sriram *et al* (9) reported that almost 95% of ameloblastomas were located in the mandible, with a very high mandible to maxilla ratio (18.1:1). This is very high compared with the ratios reported by earlier studies ( Table 5). Reichart *et al*. (49) in their extensive review of 3,677 cases of ameloblastoma, found the ratio to be around 5.4:1. In the present study, this ratio was found as 5:1.Reichart *et al* (49) also reported that ameloblastomas are seen more frequently in the anterior region among Blacks (21.6%) compared to Caucasians (12.6%) and Asians (11.9%).

**Table 5:**
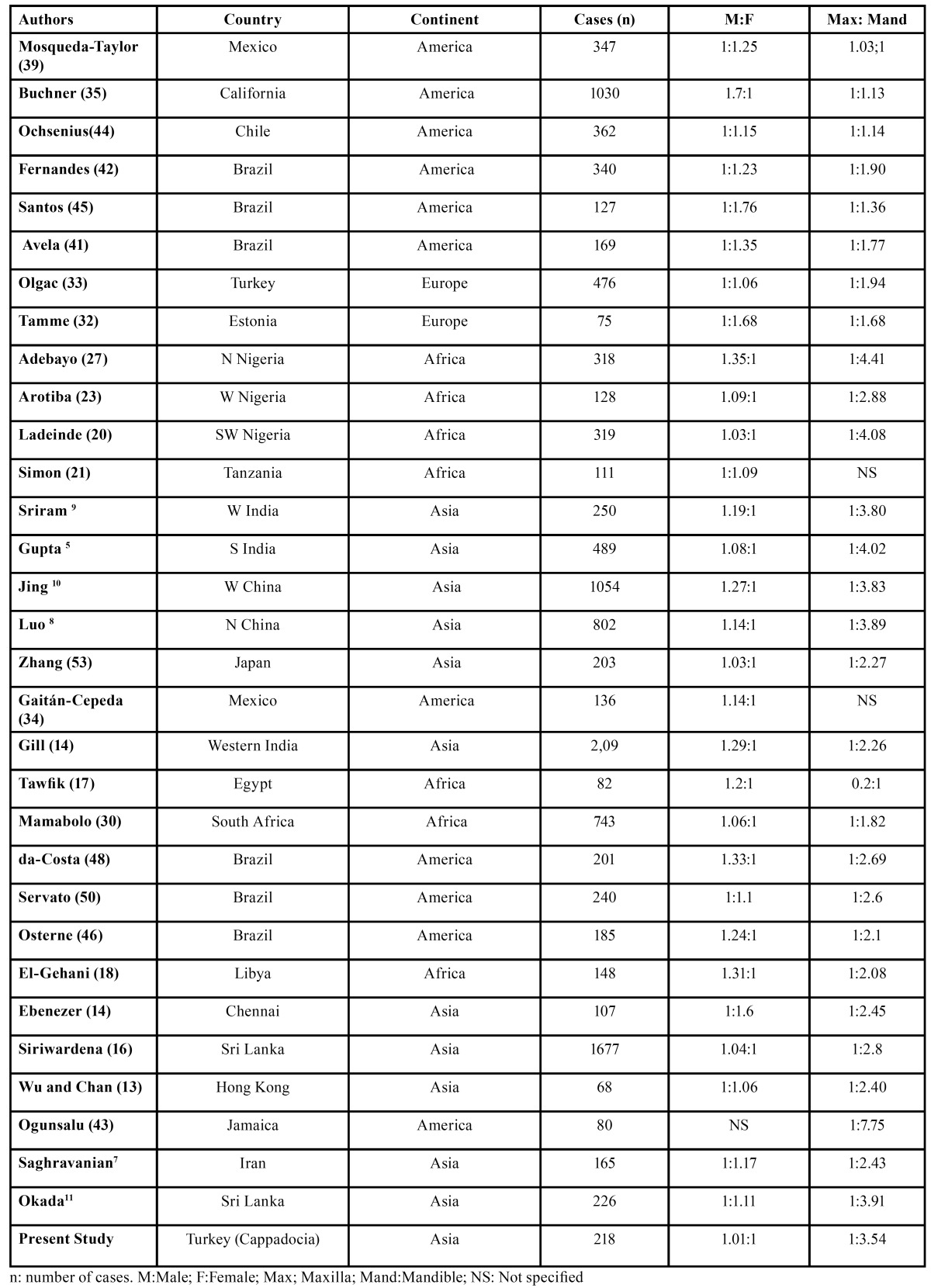
Gender and site distribution of odontogenic tumors with large studies reported from different countries and regions.

In the present series, the second most common odontogenic tumor was KCOT (26.15%) and, in accordance with other series (50-52), it was responsible for nearly a quarter of the evaluated OT. This incidence was somewhat higher (14,17,18,30), and somewhat lower (8,10,15,34,41,46,48) than that seen in other series. This study also demonstrated that KCOT is rare in early childhood and has a strikingly higher prevalence during adolescence, when it was the most common OT.

Odontomas are an abnormal mass of calcified dental tissue, usually representing a developmental abnormality. Female patients were more affected than male patients in the present study, which is in agreement with reports from China (10), whereas Ladeinde *et al*. (20) in Nigeria reported no sex predilection in their study. In the present study, most odontomas were found in the posterior regions of both jaws. This finding was in accordance with many other reports from Mexico, Chile, Brazil, and Estonia (33,39,42,44).

Reports from African and Chinese populations generally present the highest frequency of malignant OT (8,10,12,20,25), while studies from North and South America have informed rates of 1.6% and lower (34,36,39,40,42,44) except for tertiary reference centers in the United States (37), Mexico (38) and the present study. In the present study, this ratio was found in 13 cases (4.59%). Published reports also stated that malignant odontogenic tumors are rare and represent 0.1-6.1% of all tumors ( Table 3, Table 4). The mean age of 64 years for malignant tumors in the present study is higher than in other studies in southern Asia (mean 46 y) and eastern Asia (mean 41 y) (8-11,53). PIOC was the most malignant entity encountered in this analysis and represented 4.58% (10 cases) of odontogenic tumors. It was found to occur more in female patients and in the maxilla. The female predilection is in contrast to other reports (19,20).

Site distribution of odontogenic tumors with large studies reported from different countries and regions is shown in  Table 5. The majority of studies confirm the mandible as the anatomic site most frequently affected by OTs, especially by ameloblastoma and KCOT, which agrees with our findings (5,17,39,46). The preference for the mandible in this study, 1:3.52, is a mean between several studies in Nigeria and African countries (19,20,25), which provide values of 2.9 to 5.7:1, in contrast to Americans (39,41,44), and Europeans (11,32), where lower values are observed in the jaw with ratios of 1 to 2:1. These values can be explained by the prevalence of AME being far greater in African countries.

In present study, almost 83% of ameloblastomas were located in the mandible, with a very high mandible to maxilla ratio of 5:1. This is similar with the studies by Reichart *et al*. (49) who found, in an extensive review of all the cases reported in the literature, the ratio to be around 5.4:1. In the present study, ameloblastomas were frequently encountered in the molar-ramus region in the mandible and the molar region in the maxilla.

In relation to sociodemographic data, a higher proportion of males were affected with OT and the average age at diagnosis was 35 years (48). The gender distribution of odontogenic tumors in large studies reported from different countries and regions was shown in  Table 5. Avelar *et al*. (41) reported that male patients were more affected than female patients, agreeing with several studies from China (10,12), Nigeria (19,20), Egypt (17), India (9), and Canada (35). However, the preponderance of females was reported in Sri Lanka (11), Brazil (42,45), Mexico (39), Chile (44), Nigeria (25), and Estonia (32). In present study, an almost equal gender distribution was observed, with a slight predominance of males.

The literature states that patients with OT are usually diagnosed in the second to fifth decades of life (8,45), but the frequency of different lesions varies with the age of the patient. In this study, odontogenic tumors showed a peak incidence in the fourth decade of life, which was probably related to the high prevalence of AME and KCOT in this age group; there was a prevalence of odontomas in the second decade, while other studies described a high frequency of ameloblastomas and KCOT. In older patients, there is a predominance of ameloblastomas and KCOT (8,10). Some studies reported that various types of OT, including AOT, odontoma, and calcifying cystic OT (CCOT), were more frequent in the second decade of life (8,12,17).

In conclusion, the present study reflects not only differences in the distribution of odontogenic tumors but also similarities among the various population samples assessed both in Asia and around the world. These data are important to assess geographic differences in the incidence of lesions and to allow clinicians to make realistic judgments in counseling patients before biopsy about the probability of diagnosis and risks associated with nonspecific clinical or radiographic lesions. The incidences of OTs observed in the present study are similar to those in previous studies from Asia and Africa and in contrast to those reported from American and European countries.
